# 25-hydroxyvitamin D_3_ generates immunomodulatory plasticity in human periodontal ligament-derived mesenchymal stromal cells that is inflammatory context-dependent

**DOI:** 10.3389/fimmu.2023.1100041

**Published:** 2023-01-24

**Authors:** Christian Behm, Alice Blufstein, Johannes Gahn, Andreas Moritz, Xiaohui Rausch-Fan, Oleh Andrukhov

**Affiliations:** ^1^ Competence Center Periodontal Research, University Clinic of Dentistry, Medical University of Vienna, Vienna, Austria; ^2^ Clinical Division of Conservative Dentistry and Periodontology, University Clinic of Dentistry, Medical University of Vienna, Vienna, Austria; ^3^ Center for Clinical Research, University Clinic of Dentistry, Medical University of Vienna, Vienna, Austria

**Keywords:** 25-hydroxyvitamin D_3_, mesenchymal stromal cells, periodontal ligament, immunomodulation, tumor necrosis factor alpha, interleukin-1 beta

## Abstract

**Introduction:**

Human periodontal ligament-derived mesenchymal stromal cells (hPDL-MSCs) exhibit a tight bi-directional interaction with CD4^+^ T lymphocytes. The hPDL-MSCs’ immunomodulatory abilities are drastically enhanced by pro-inflammatory cytokines via boosting the expression of various immunomediators. 25-hydroxyvitamin D_3_ (25(OH)D_3_), the major metabolite of vitamin D3 in the blood, affects both hPDL-MSCs and CD4^+^ T lymphocytes, but its influence on their interaction is unknown.

**Methods:**

Therefore, primary hPDL-MSCs were stimulated *in vitro* with tumor necrosis factor (TNF)-α a or interleukin (IL)-1β in the absence and presence of 25(OH)D_3_ followed by an indirect co-culture with phytohemagglutinin-activated CD4^+^ T lymphocytes. The CD4^+^ T lymphocyte proliferation, viability, and cytokine secretion were analyzed. Additionally, the expression of various immunomediators in hPDL-MSCs was investigated, and their implication was verified by using pharmacological inhibitors.

**Results:**

25(OH)D_3_ significantly counteracted the suppressive effects of IL-1β-treated hPDL-MSCs on CD4^+^ T lymphocyte proliferation, whereas no effects were observed in the presence of TNF-α. Additionally, 25(OH)D_3_ significantly increased the percentage of viable CD4^+^ T lymphocytes via TNF-α- or IL-1β-treated hPDL-MSCs. It also caused a significant decrease in interferon-γ, IL-17A, and transforming growth factor-β productions, which were triggered by TNF-α-treated hPDL-MSCs. 25(OH)D_3_ significantly decreased the production of various immunomediators in hPDL-MSCs. Inhibition of two of them, prostaglandin E2 and indoleamine-2,3-dioxygenase-1, partially abolished some of the hPDL-MSCs-mediated effects of 25(OH)D_3_ on CD4^+^ T lymphocytes.

**Conclusion:**

These data indicate that 25(OH)D_3_ influences the immunomodulatory activities of hPDL-MSCs. This modulatory potential seems to have high plasticity depending on the local cytokine conditions and may be involved in regulating periodontal tissue inflammatory processes.

## 1 Introduction

Mesenchymal stromal cells (MSCs) are a non-hematopoietic, heterogenous cell population that is characterized by the expression of specific surface markers, self-renewing, and multipotent differentiation potential *in vitro* (1–3). MSCs can be isolated from various tissues throughout the human body, such as the umbilical cord, bone marrow, and adipose tissue (4, 5). Another source for MSCs is dental tissues, such as the periodontal ligament (PDL) (6), which is a highly specialized connective tissue of the periodontium connecting the teeth’s roots to the alveolar bone (7). Like MSCs from other sources, human periodontal ligament-derived MSCs (hPDL-MSCs) are key components to keep up local tissue homeostasis and regulate tissue regeneration and inflammation (4, 8, 9). Under homeostatic conditions, hPDL-MSCs are in a quiescent and undifferentiated state located in the perivascular niche (10, 11). hPDL-MSCs are activated by periodontal tissue injury or inflammation and migrate to the injured tissue site through chemotaxis-driven mechanisms. At the injury site, hPDL-MSCs are involved in restoring periodontal tissue homeostasis, which is mainly accomplished by modulating the local immune response (4, 8, 9).

The immunomodulatory activities of hPDL-MSCs are mainly suppressive against cells of the innate and adaptive immune system. Multiple studies have already demonstrated that hPDL-MSCs inhibit the proliferation of CD4^+^ T lymphocytes and their differentiation to pro-inflammatory subsets and favor the formation of regulatory CD4^+^ T lymphocytes (T_regs_) (4, 5). These immunomodulatory activities of hPDL-MSCs are mediated through the production of specific immunomediators. Soluble immunomediators, such as indoleamine-2,3-dioxygenase-1 (IDO-1), prostaglandin E_2_ (PGE_2_) and tumor necrosis factor-inducible gene 6 (TSG-6), and membrane-bound factors, like programmed cell death 1 ligand 1 (PD-L1) and programmed cell death 1 ligand 2 (PD-L2), enable an interaction between hPDL-MSCs and immune cells *via* paracrine and direct cell-to-cell contact mechanisms, respectively (4, 12). Usually, these mechanisms are low at homeostatic conditions and are boosted by various environmental factors, like immune cell-derived cytokines, such as tumor necrosis factor alpha (TNF-α) or interleukin- (IL-) 1β (13, 14). This causes a tight bi-directional interplay between hPDL-MSCs and immune cells (5, 15), and the character of this interplay is dictated by the cytokine environment (14).

The fat-soluble secosteroid hormone vitamin D_3_ is another well-known factor affecting local immune responses, showing anti-inflammatory properties (16, 17). However, data from clinical studies are inconsistent concerning its beneficial effect on inflammatory diseases. Although the severity of various inflammatory diseases is associated with vitamin D_3_ deficiency (18, 19), dietary supplementation has no apparent positive impact on different conditions (20, 21). Regarding periodontitis, which is a highly prevalent, chronic inflammatory disease of the tooth-supporting tissues causing tooth loss (22), the clinical data on vitamin D_3_ are imperfect. Most clinical studies showed an association between vitamin D_3_ deficiency and the incidence and severity of periodontitis (23–25), but one study demonstrated a positive relationship between increased vitamin D_3_ serum levels and the incidence and severity of periodontitis (26). Moreover, the data on the effect of dietary supplementation of vitamin D_3_ during periodontitis therapy are also inconsistent (27, 28). These contradictory results indicate that the functions of vitamin D_3_ in the periodontium may be complex and versatile and may not be considered as exclusively anti-inflammatory. Hence it is essential to take a closer look at the immunomodulating activities of various vitamin D_3_ forms. Like the biologically active form of vitamin D_3_, 1,25-dihydroxyvitamin D_3_ (1,25(OH)_2_D_3_), its metabolic precursor 25-hydroxyvitamin D_3_ (25(OH)D_3_) (29, 30) shows anti-inflammatory properties (31–35), attenuating experimental periodontitis in various animal models (36–38). Our previous study also demonstrated that 25(OH)D_3_ decreases the production of several pro-inflammatory cytokines by hPDL-MSCs in response to bacterial stimuli (31). However, no study exists that investigates the ability of 25(OH)D_3_ to influence the immunomodulatory activities of hPDL-MSCs and their reciprocal interaction with immune cells.

Hence, the main objective of this *in vitro* study was to investigate a potential indirect influence of 25(OH)D_3_ on CD4^+^ T lymphocytes in the presence of cytokine-treated hPDL-MSCs using an indirect co-culture model. In particular, we examined the proliferation, viability, and secretion of cytokines specific to the various CD4^+^ T lymphocyte subsets. Co-cultured hPDL-MSCs were treated with either TNF-α or IL-1β to figure out the potential different effects of 25(OH)D_3_ depending on the cytokine milieu. Additionally, we investigated and directly compared the influence of 25(OH)D_3_ on the TNF-α- or IL-1β-induced expression of various immunomediators by hPDL-MSCs and verified their involvement in the hPDL-MSCs mediated effects of 25(OH)D_3_ on CD4^+^ T lymphocytes using pharmacological inhibitors. Our data indicate that 25(OH)D_3_ affects the immunomodulatory mechanisms of hPDL-MSCs against CD4^+^ T lymphocytes. This effect shows high plasticity and is largely determined by the cytokine context.

## 2 Material and Methods

### 2.1 Ethical approval

The study was reviewed and approved by the Ethics Committee of the Medical University of Vienna (EK Nr.: 1694/2015, extended up to 10/2023). All subsequent experiments were undertaken following the Declaration of Helsinki and the Good Scientific Practice Guidelines of the Medical University of Vienna.

### 2.2 Cell isolation

#### 2.2.1 Primary hPDL-MSCs isolation

Third molars were donated from 10 periodontally healthy patients, aged between 15 to 27, who underwent tooth extraction due to orthodontic reasons. All patients got informed before the surgical procedure and gave their written consent. The mid-third of the tooth’s root was used to scrap off PDL slices, which were minced and cultured at 37° Celsius, 5% carbon dioxide, and 95% humidity using Dulbecco’s modified Eagle’s medium (DMEM, Sigma-Aldrich, St. Louis, USA) supplemented with 10% fetal bovine serum (FBS, Gibco, Carlsbad, USA), 100U/ml penicillin and 50µg/ml streptomycin (Gibco, Carlsbad, USA). After the cells had grown out from the tissue explants, hPDL-MSCs were subcultured under the same conditions described above. When reaching 80% confluency, passages three to seven of hPDL-MSCs were used for all conducted experiments. Since it is well known that primary MSCs show high inter-donor variations (39, 40), hPDL-MSCs from different patients were used to consider this variability.

All used hPDL-MSCs conformed to the minimal criteria for MSCs specified by the International Society for Cell and Gene Therapy (3). The expression of mesenchymal stem/stromal (CD29, CD90, CD105, CD146) and hematopoietic (CD14, CD31, CD34, CD45) surface markers was verified as previously described (14, 41).

#### 2.2.2 Human CD4^+^ T lymphocyte isolation

Whole blood was collected from the median cubital or cephalic vein using the heparin- and lithium-containing VACUETTE^®^ blood collection system (Greiner Bio-one, Kremsmünster, Austria). Blood was taken only from one volunteer throughout the whole study to exclude potential CD4^+^ T lymphocyte donor-to-donor variability. This allows focusing only on the hPDL-MSCs-based inter-individual variations since this study aimed to evaluate hPDL-MSCs-mediated effects of 25(OH)D_3_. From the collected blood peripheral blood mononuclear cells (PBMCs) were isolated. In brief, donated blood was mixed 1:1 with Hank’s Balanced Salt Solution (HBSS, Life Technologies, Carlsbad, USA) followed by Ficoll-Paque (GE Healthcare, Chicago, USA) density gradient centrifugation. After harvesting and washing the PBMCs, the MagniSort™ Human CD4^+^ T cell enrichment kit (Invitrogen, Carlsbad, USA) was used for the immunomagnetic negative selection of CD4^+^ T lymphocytes using 1x phosphate buffered saline (1xPBS). The purity of enriched CD4^+^ T lymphocytes (93%) was verified in our previous study by CD4 immunostaining followed by flow cytometry analysis (42).

### 2.3 Cell treatment

#### 2.3.1 hPDL-MSCs/CD4^+^ T lymphocyte indirect co-culture

**Figure 1:**
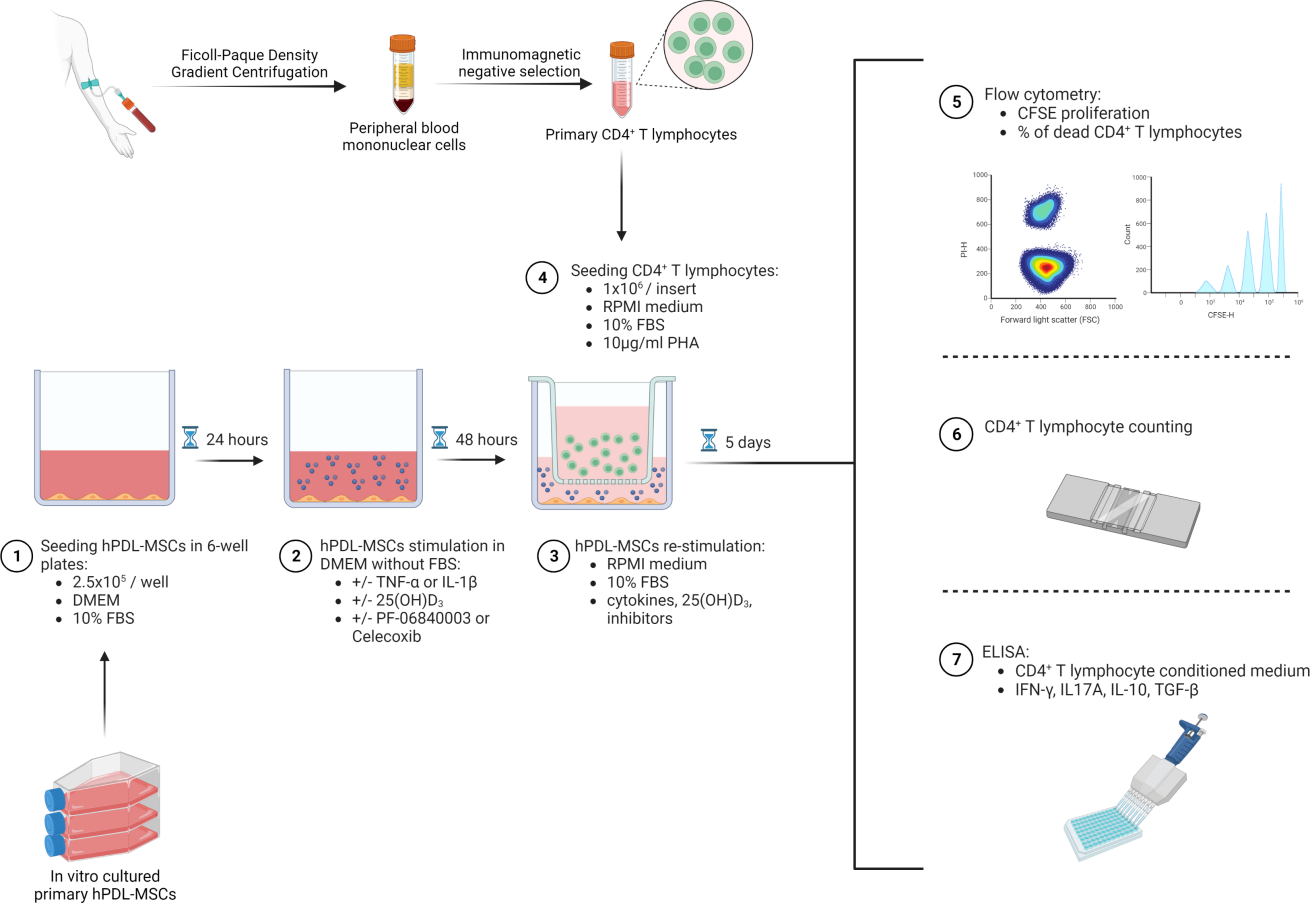
Graphical presentation and timeline of the indirect co-culture experimental setup. Created with BioRender.

2.5x10^5^ primary hPDL-MSCs were cultured per well in 6-well plates using 3ml DMEM supplemented with 10% FBS, 100U/ml penicillin, and 50µg/ml streptomycin (**Figure 1**). After 24 hours of incubation, hPDL-MSCs were pre-stimulated with 10ng/ml TNF-α (43) or 5ng/ml IL-1β (44) (both from *In vivo*gen, San Diego, USA) in combination with 100nM 25(OH)D_3_ (Cayman Chemical, Ann Arbor, USA). Untreated, as well as cytokines solely treated hPDL-MSCs, served as control. This pre-stimulation procedure was performed under FBS-free conditions. After 48 hours of incubation, hPDL-MSCs were re-stimulated, as described above, with cytokines and 25(OH)D_3_ using RPMI medium (Sigma-Aldrich, St. Louis, USA), supplemented with 10% FBS, 100U/ml penicillin, and 50µg/ml streptomycin. Per group, 1x10^6^ freshly isolated, allogeneic CD4^+^ T lymphocytes were indirectly added to hPDL-MSCs using Transwell (TC) inserts with 0.4µm pore size (Sarstedt, Nürnbrecht, Germany), and stimulated with phytohemagglutinin-L (PHA-L, 10µg/ml, eBioscience, San Diego, USA) to induce their proliferation. PHA-L stimulated CD4^+^ T lymphocytes in the absence of hPDL-MSCs, but in the presence of various cytokines and 25(OH)D_3_, served as a reference. After five days of incubation, the proliferation and the percentage of non-viable CD4^+^ T lymphocytes were determined by carboxyfluorescein succinimidyl ester (CFSE) and propidium iodide (PI, both from Thermo Fisher Scientific, Waltham, USA) staining, respectively, followed by flow cytometry analysis. Additionally, interferon (IFN)-γ, IL-17A, IL-4, IL-10, and transforming growth factor (TGF)-β levels were determined in conditioned media using appropriate enzyme-linked immunosorbent assays.

In one run of experiments, either 1µM PTGS-2 inhibitor (Celecoxib) (45) or 50µM IDO-1 inhibitor (PF-06840003) (14) were added to hPDL-MSCs during the pre-stimulation and co-culture (both from Selleck Chemicals, Houston, USA) period. Analysis was performed as described above.

#### 2.3.2 hPDL-MSCs treatment for immunomediators expression analysis

2.5x10^5^ primary hPDL-MSCs were seeded per well in 6-well plates using 3ml DMEM supplemented with 10% FBS, 100U/ml penicillin, and 50µg/ml streptomycin. After 24 hours of incubation, hPDL-MSCs were stimulated with different cytokines in the absence or presence of 25(OH)D_3_ as described above. Immunomediator expression was analyzed after 48 hours of incubation using quantitative polymerase chain reaction (qPCR), immunostaining followed by flow cytometry analysis, ELISA, and enzymatic activity analysis.

### 2.4 Analysis

#### 2.4.1 Proliferation and the percentage of non-viable CD4^+^ T lymphocytes

CD4^+^ T lymphocyte proliferation was assessed using CellTrace CFSE Cell Proliferation Kit (ThermoFisher Scientific, Waltham, USA) according to the manufacturer’s instructions. In brief, 1x10^6^ CD4^+^ T lymphocytes per ml were resuspended in 1xPBS containing 5% FBS and stained with 2.5µM CFSE. After five minutes of incubation at room temperature, washing steps, and a recovery period, labeled CD4^+^ T lymphocytes were added to the indirect co-culture model using a complete RPMI medium. After five days of incubation, CD4^+^ T lymphocytes were harvested and washed using 3% bovine serum albumin (BSA, Merck Millipore, Burlington, USA). Additionally, CD4^+^ T lymphocytes were stained with 20µg/ml propidium iodide (PI, Affymetrix, Santa Clara, USA) to determine the percentage of non-viable CD4^+^ T lymphocytes and to exclude them from the proliferation analysis. Both CD4^+^ T lymphocyte proliferation and the percentage of non-viable cells were analyzed by an Attune NxT flow cytometer (ThermoFisher Scientific, Waltham, USA). CFSE, as well as PI fluorescence, was excited at 488nm, and fluorescence emissions were detected at the BL1 and BL2 channels, respectively. Non-proliferating unlabeled, CFSE-, or PI single-labeled CD4^+^ T lymphocytes were used for compensation. In all three controls living CD4^+^ T lymphocytes were mixed with dead cells (1:1). In total, 20,000 CD4^+^ T lymphocytes were acquired per sample. After excluding coincidence events and dead cells (for the gating strategy, see **Supplementary Figure 1**), the percentage of original CD4^+^ T lymphocytes that undergone cell division, the number of original CD4^+^ T lymphocytes as a percent of the total number of original cells (for each CD4^+^ T lymphocyte generation), and the percentage of PI^+^ CD4^+^ T lymphocytes were determined using FCS Express 7 (*De Novo* Software, Pasadena, USA).

#### 2.4.2 CD4^+^ T lymphocyte cytokine production

IFN-γ, IL-17A, IL-10, and TGF-β were detected in conditioned media, which were harvested after five days of indirect co-culture. Cytokine concentrations were determined using IFN gamma Human Uncoated, IL-17A (homodimer) Human Uncoated, IL-10 Human Uncoated (all from ThermoFisher Scientific, Waltham, USA), and Human TGF-beta 1 ELISA kit (RayBiotech, Peachtree Corners, USA) in accordance to the manufacturer’s instructions. In brief, cytokine levels were evaluated photometrically by measuring absorbance at OD_450_ (optical density) using a microplate reader (Synergy HTX multiplate reader, BioTek Winooski, USA). For IFN-γ, IL-17A, and IL-10, absorbance was additionally evaluated at OD_570_, followed by plotting OD_570_-substracted values against the appropriate four-parameter logistic regression. For TGF-β, OD_450_ values were directly plotted against the corresponding polynomial regression. Standard curves ranged between 4-500pg/ml for IFN-γ and IL-17A and between 2-300pg/ml and 18-4000pg/ml for IL-10 and TGF-β, respectively. IL-4 was beneath the detection limit using IL-4 Human Uncoated ELISA (ThermoFisher Scientific, Waltham USA) with an assay range between 2-200pg/ml (data not shown). Determined cytokine concentrations of every single sample were normalized to the corresponding total number of CD4^+^ T lymphocytes, which was evaluated by using a Neubauer Improved Cell counting chamber (NanoEnTek, Soul, South Korea) after five days of indirect co-culture.

#### 2.4.3 Immunomediators’ gene expression analysis

After 48 hours of treating hPDL-MSCs with various cytokines and 25(OH)D_3_ in the absence of CD4^+^ T lymphocytes, IDO-1, TSG-6, PTGS-2, PD-L1, and PD-L2 gene expression levels were determined using TaqMan Gene Expression Cells-to-CT kit (Applied Biosystems, Foster City, USA) in accordance to the manufacturer’s instructions. After cell lysis, mRNA was reversely transcribed to cDNA by heating samples to 37° Celsius for one hour and to 95° Celsius for five minutes using the Primus 96 advanced thermocycler (PeqLab/VWR, Darmstadt, Germany). For quantification of gene expression, cDNA was heated once to 95° Celsius for ten minutes and 50x to 95° Celsius for 15 seconds, and to 60° Celsius for 1 minute using the QuantStudio 3 device (Applied Biosystems, Foster City, USA). The following TaqMan Gene Expression Assays (Applied Biosystems, Foster City, USA) were used to specifically amplify target genes: IDO-1, Hs00984148_m1; TSG-6, Hs00200180_m1; PTGS-2, Hs00153133_m1; PD-L1, Hs00204257_m1; PD-L2, Hs00228839_m1 and GAPDH, Hs99999905_m1. The housekeeping gene glyceraldehyde-3-phosphate dehydrogenase (GAPDH) served as an internal loading control. After amplifying target genes in paired reactions and determining the GAPDH-normalized C_t_ values (ΔCt), the n-fold gene expression compared to the unstimulated control (n-fold expression = 1) was calculated using the 2^-ΔΔCt^ method.

#### 2.4.4 Intracellular IDO-1 protein expression analysis

After fixing and permeabilization using the Intracellular Fixation and Permeabilization Buffer Set (eBioscience, Waltham, USA), 2.5x10^5^ hPDL-MSCs were intracellularly stained by 0.06µg of the R-phycoerythrin (R-PE)-conjugated mouse anti-human IDO-1 monoclonal (clone eyedio) antibody (Thermo Fisher Scientific, Waltham, USA) for 30 minutes. Additionally, hPDL-MSCs were labeled with PE-conjugated mouse IgG1 κ immunoglobulin isotype control (Thermo Fisher Scientific, Waltham, USA) which served as control. Subsequently, cells were washed several times and resuspended in 0.5ml 3% bovine serum albumin (BSA, Capricorn Scientific, Ebsdorfergrund, Germany, in 1xPBS + 0.09% sodium azide, Merck Darmstadt, Germany) for acquisition. Flow cytometry analysis was performed using FACSCalibur Flow Cytometer (BD Biosciences, Franklin Lakes, USA) exciting R-PE by an argon laser at 488nm. In total, 10,000 cells were acquired per sample. Gates were set using unlabeled hPDL-MSCs. The gating strategy is described in the **Supplementary Material** of our previous study (2). The percentage of IDO-1^+^ hPDL-MSCs and the corresponding mean fluorescence intensity (M.F.I.) were calculated by CellQuest 3.3. software (BD Bioscience, Franklin Lakes, USA).

#### 2.4.5 IDO-1 enzymatic activity analysis

IDO-1 enzymatic activity was analyzed by determining L-kynurenine concentrations in conditioned media from hPDL-MSCs. Briefly, the conditioned medium was diluted 1:3 (v/v) with 30% trichloroacetic acid (Sigma-Aldrich, St. Louis, USA), which was followed by incubating samples for 30 minutes at 65° Celsius. After centrifugation, 125µl Ehrlich’s Reagent, consisting of 0.8% P-dimethylbenzaldehyde in glacial acetic acid (Sigma-Aldrich, St. Louis, USA), was added 1:1 to the supernatant. After 10 minutes of incubation at room temperature, absorbance was measured in duplicates at 492nm (OD_492_) using a microplate reader (Synergy HTX multiplate reader, BioTek, Winooski, USA). L-kynurenine concentrations were calculated by plotting measured OD_492_ against a linear regression curve with L-kynurenine (Sigma-Aldrich, St. Louis, USA) concentrations ranging from 7.8µM to 1000µM. The calculated L-kynurenine concentrations were normalized to the total protein amount and the unstimulated control (= 0). The Pierce BCA Protein Assay Kit (Thermo Fisher Scientific, Waltham, USA) was used according to the manufacturer’s instructions to determine the total protein amount in mg. In brief, quantified absorbance at 562nm (OD_562_) was plotted against a linear regression curve composed of known BSA (Capricorn Scientific, Ebsdorfergrund, Germany) concentrations which ranged between 31.25µg/ml and 2000µg/ml.

#### 2.4.6 TSG-6 protein expression and PGE_2_ production analysis

TSG-6 and PGE_2_ concentrations were determined in the harvested conditioned media using TSG-6 ELISA (RayBiotech, Peachtree Corners, USA) and Prostaglandin E_2_ Parameter Assay Kit (R&D Systems, Minneapolis, USA), respectively. According to the manufacturer’s instructions, optical density was measured at 450nm (OD_450_) using the microplate reader (Synergy HTX multiplate reader, BioTek, Winooski, USA). For TSG-6, measured absorbance values were directly plotted against the standard curve ranging between 0.2ng/ml and 50ng/ml. For PGE_2_, measured OD_540_ values were subtracted from the corresponding OD_450_ values and plotted against the standard curve. The detection range of the PGE_2_ standard curve was between 30pg/ml and 2500pg/ml.

#### 2.4.7 PD-L1 and PD-L2 protein expression analysis

After harvesting and several wash steps, 2.5x10^5^ hPDL-MSCs were resuspended in 3% BSA solution (in 1xPBS + 0.09% sodium azide) and stained either with 0.5µg R-PE-conjugated mouse anti-human CD274 (PD-L1) monoclonal (clone B7-H1) antibody or with 0.125µg R-PE-conjugated mouse anti-human CD273 (PD-L2) monoclonal (clone B7-DC) antibody (both from Thermo Fisher Scientific, Waltham, USA). hPDL-MSCs labeled with PE-conjugated mouse IgG1κ immunoglobulin isotype control (Thermo Fischer Scientific, Waltham, USA) served as control. After 30 minutes of incubation at room temperature and several wash steps, hPDL-MSCs were resuspended in 0.5ml 3% BSA solution. In total, 10,000 hPDL-MSCs were acquired per group using the FACSCalibur Flow Cytometer (BD Biosciences, Franklin Lakes, USA). R-PE was excited by an argon laser at 488nm, and the gates were placed depending on an unlabeled control. The exact gating strategy is described in our previous work under the **Supplementary Section** (14). The CellQuest 3.3. software (BD Bioscience, Franklin Lakes, USA) was used to determine the % of PD-L1^+^ or PD-L2^+^ hPDL-MSCs and to calculate the appropriate M.F.I.

### 2.5 Statistical Analysis

All statistical analysis was conducted using SPSS Statistics 24.0 software (IBM, Armonk, USA). Normal distribution was verified by Kolmogorov-Smirnov test. Non-parametric data were analyzed using Friedman test, followed by Wilcoxon test for pairwise comparison, whereas normally distributed data were evaluated using one-way ANOVA and Tukey’s multiple comparison test. P-values < 0.05 were considered to be statistically significant. All data were obtained from at least four experimental repetitions using hPDL-MSCs from a different patient per repetition.

## 3 Results

### 3.1 The cytokine type dictates the hPDL-MSCs mediated effects of 25(OH)D_3_ on CD4^+^ T lymphocytes proliferation and viability

**Figure 2:**
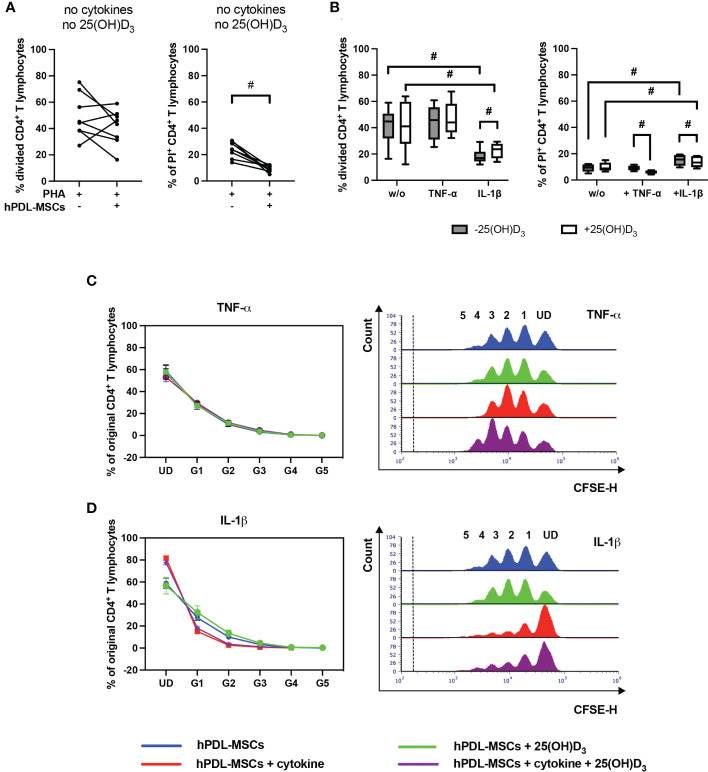
25(OH)D_3_ differently affects CD4^+^ T lymphocyte proliferation and viability *via* hPDL-MSCs depending on the present cytokine. After five days of culturing CD4^+^ T lymphocytes indirectly with hPDL-MSCs, flow cytometry analysis was used to determine the proliferation and viability of CD4^+^ T lymphocytes by CFSE and PI staining, respectively. **(A, B)** reveals the percentage of original CD4^+^ T lymphocytes that are divided and the percentage of PI^+^ CD4^+^ T lymphocytes. **(C, D)** shows the number of original CD4^+^ T lymphocytes as a percent of the total number of original cells for each cell generation (UD = undivided cells, G1-G5 = generation 1 – generation 5). Additionally, **(C, D)** includes representative histograms showing the number of CD4^+^ T lymphocytes for all generations (UD and 1-5). Data were obtained from five **(B, C, D)** and eight **(A)** experimental repetitions using hPDL-MSCs from a different individual per repetition. One technical replicate was used per experimental group. In **(A)** the percentage of divided and PI^+^ CD4^+^ T lymphocytes are represented as an individual line for each experimental repetition, whereas in **(B)** data are presented as a box-whisker-plot showing the minimum and maximum values. The percentage of original CD4^+^ T lymphocytes in **(C, D)** are presented as mean ± standard error of the mean (S.E.M.). The Friedman test, followed by the Wilcoxon test, was used for pairwise comparison. # p-value < 0.05 were considered to be statistically significant between groups as indicated.

**Figure 2** shows the effect of 25(OH)D_3_ on the proliferation (**Figures 2B, C–D**) and viability (**Figure 2B**) of CD4^+^ T lymphocytes in the indirect co-culture model. hPDL-MSCs alone had no significant effect on CD4^+^ T lymphocyte proliferation but exerted an anti-cell-death effect (**Figure 2A**). In the absence of any cytokine, 25(OH)D_3_ had no influence on hPDL-MSCs-mediated effects on CD4^+^ T lymphocytes (**Figure 2B**). Only IL-1β-treated hPDL-MSCs significantly inhibited CD4^+^ T lymphocyte proliferation (**Figure 2B**), showing a decreased % of original CD4^+^ T lymphocytes in the formed generations 1-4 (G1-G4) (**Figure 2D**). Additionally, IL-1β significantly counteracted the anti-cell-death effect of hPDL-MSCs (**Figure 2B**). These effects of IL-1β-treated hPDL-MSCs on the proliferation and viability of CD4^+^ T lymphocytes were counteracted by 25(OH)D_3_, although the percentage of divided and PI^+^ CD4^+^ T lymphocytes was still significantly lower and higher compared to non-inflammatory conditions, respectively (**Figure 2B**). In the presence of TNF-α treated hPDL-MSCs, 25(OH)D_3_ significantly reduced the percentage of non-viable CD4^+^ T lymphocytes (**Figure 2B**). These data indicate that the effects of 25(OH)D_3_ on the interaction between hPDL-MSCs and CD4^+^ T lymphocytes depends on the cytokine type and seems to be more pro-inflammatory in the presence of IL-1β.

### 3.2 The cytokine type dictates the hPDL-MSCs mediated effects of 25(OH)D_3_ on the cytokine production in CD4^+^ T lymphocyte

**Figure 3:**
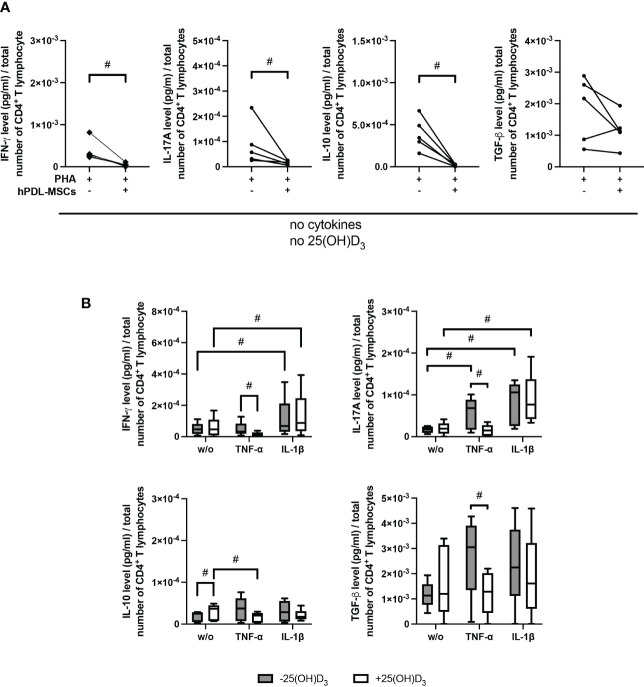
25(OH)D_3_ differently affects the production of IFN-γ, IL-17A, IL-10, and TGF-β in CD4^+^ T lymphocytes *via* hPDL-MSs treated with various pro-inflammatory cytokines. After five days of culturing CD4^+^ T lymphocytes indirectly with hPDL-MSCs, the concentration of Th1 (IFN-γ), Th17 (IL-17A), and regulatory T lymphocytes (IL-10, TGF-β) cytokines were measured in conditioned media using appropriate enzyme-linked immunosorbent assays (ELISA) and the total CD4^+^ T lymphocyte numbers per sample were determined using a cell counting chamber. Measured cytokine levels (pg/ml) were normalized to the total CD4^+^ T lymphocyte number. Data were obtained from five **(A, B)** experimental repetitions using hPDL-MSCs from a different individual per repetition. One technical replicate was used per experimental group. In **(A)** the normalized cytokine levels are represented as an individual line for each experimental repetition, whereas in **(B)** data are presented as a box-whisker-plot showing the minimum and maximum values. The Friedman test, followed by the Wilcoxon test, was used for pairwise comparison. # p-value < 0.05 were considered to be statistically significant between groups as indicated.

**Figure 3** demonstrates the effect of 25(OH)D_3_ on the production of different Th subsets-associated cytokines in the indirect co-culture model. hPDL-MSCs alone significantly inhibited the production of IFN-γ, IL-17A and IL-10 (**Figure 3A**). The hPDL-MSCs mediated decrease in IL-10 was counteracted by 25(OH)D_3_ (**Figure 3B**). Adding any cytokine in the absence of 25(OH)D_3_ counteracted the hPDL-MSCs-mediated inhibition of IL-17A (**Figure 3B**). In addition, IL-1β-treated hPDL-MSCs caused a significant increase in IFN-γ production (**Figure 3B**). Adding 25(OH)D_3_ to TNF-α-triggered hPDL-MSCs resulted in the significant inhibition of IFN-γ, IL-17A and TGF-β production (**Figure 3B**). In the presence of IL-1β, 25(OH)D_3_ had no significant effect on any cytokine production (**Figure 3B**). These data suggest a dependency of the hPDL-MSCs-mediated effects of 25(OH)D_3_ on the local cytokine milieu, showing the potential to reduce the production/secretion of pro- and anti-inflammatory cytokines in CD4^+^ T lymphocytes in the presence of TNF-α-treated hPDL-MSCs.

### 3.3 25(OH)D_3_ decreases gene and protein expression of soluble and membrane-bound immunomediators in hPDL-MSCs

**Figure 4:**
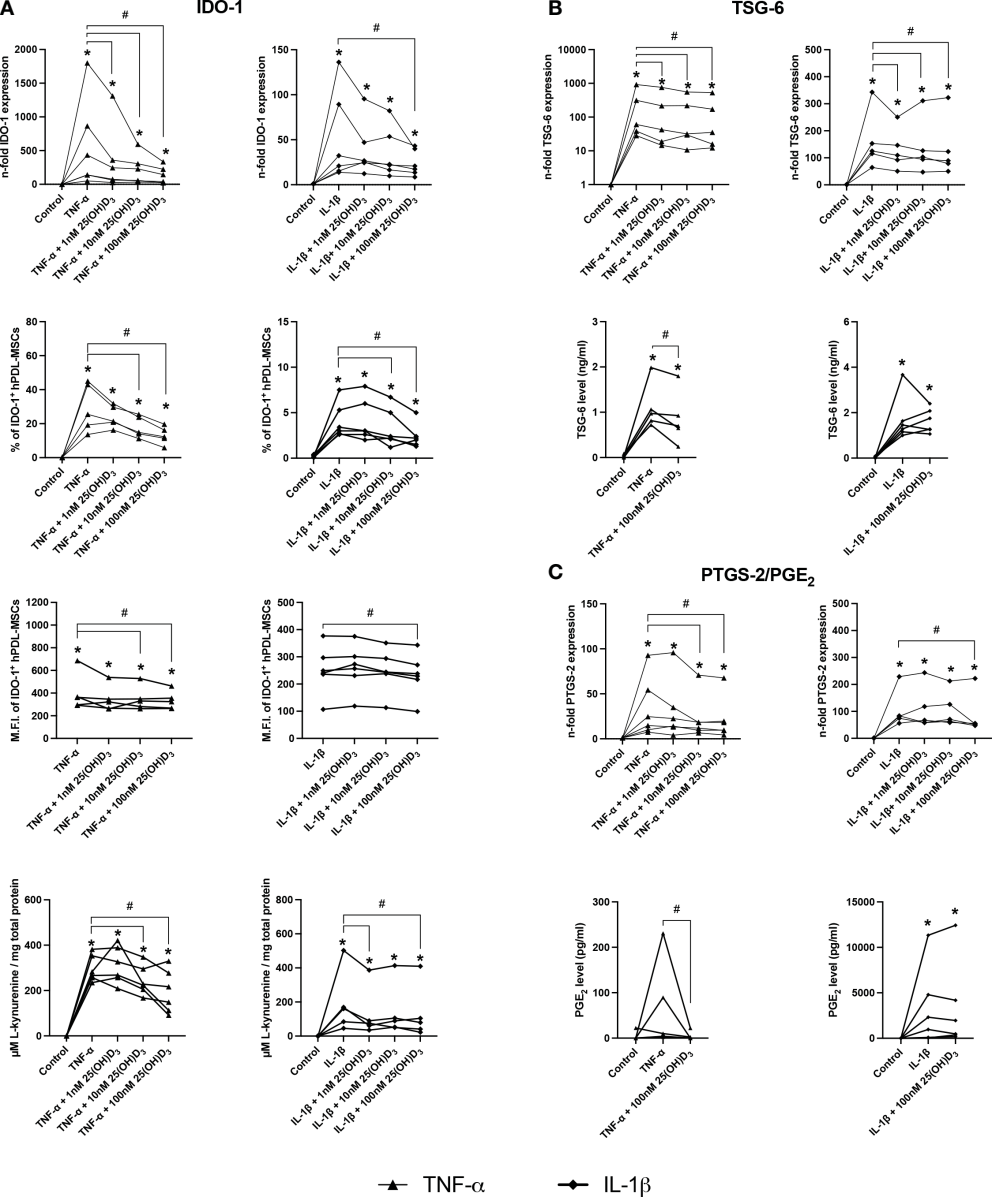
25(OH)D_3_ decreases TNF-α and IL-1β induced expression of soluble immunomediators in hPDL-MSCs. After 48 hours of incubation, the expression of IDO-1 **(A)**, TSG-6 **(B)**, and PTGS-2/PGE_2_ **(C)** were investigated on gene and protein levels. Gene expression levels were determined by qPCR (A, B and C) followed by calculating the n-fold gene expression compared to unstimulated hPDL-MSCs (n-fold expression = 1). GAPDH served as an internal reference. IDO-1 protein expression **(A)** was detected by intracellular immunostaining followed by flow cytometry, calculating the percentage of IDO-1^+^ hPDL-MSCs and the corresponding mean fluorescence intensity (M.F.I.). TSG-6 **(B)** and PGE_2_ **(C)** levels in conditioned media were determined by ELISA in ng/ml and pg/ml, respectively. Additionally, IDO-1 enzymatic activity **(A)** was determined by measuring L-kynurenine level (µM) in conditioned media photometrically, which was normalized to the total protein amount (mg) per group and the unstimulated control. Data were obtained from five or six **(A–C)** experimental repetitions using hPDL-MSCs from a different individual per repetition. For qPCR **(A-C)** and IDO-1 enzymatic activity assay **(A)** two technical replicates were used, whereas one technical replicate was used per experimental group for IDO-1 immunostaining **(A)**, TSG-6 **(B)** and PGE_2_ **(C)** ELISA. Each experimental repetition is represented as an individual line **(A–C)**. The Friedman test, followed by the Wilcoxon test, was used for pairwise comparison. * p-value < 0.05 were considered to be significantly increased compared to unstimulated hPDL-MSCs. # p-value < 0.05 were considered to be significantly decreased compared to cytokine-stimulated hPDL-MSCs in the absence of 25(OH)D_3_.

**Figure 5:**
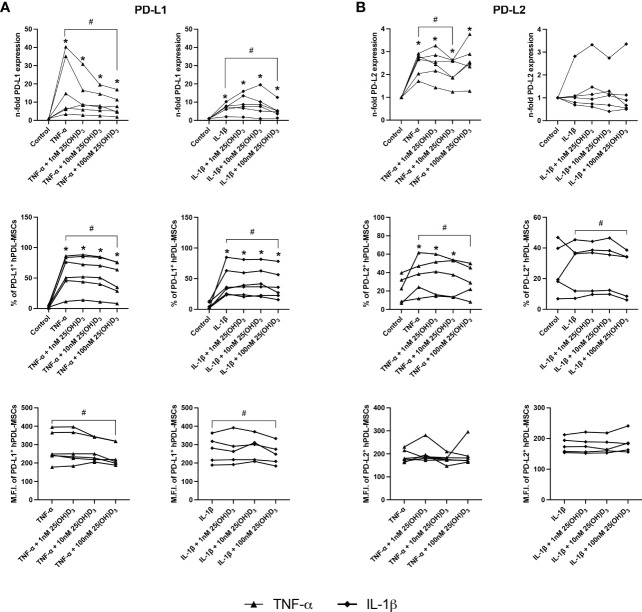
25(OH)D_3_ decreases the TNF-α and IL-1β induced expression of membrane-bound immunomediators in hPDL-MSCs. After 48 hours of incubation, the expression of PD-L1 **(A)** and PD-L2 **(B)** were determined on gene and protein levels. Gene expression levels were determined by qPCR **(A, B)** followed by calculating the n-f old expression compared to unstimulated hPDL-MSCs (n-fold expression = 1). GAPDH served as an internal reference. PD-L1 **(A)** and PD-L2 **(B)** protein expression were determined by surface marker immunostaining followed by flow cytometry calculating the percentage of PD-L1^+^ and PD-L2^+^ hPDL-MSCs and the corresponding M.F.I. Data were obtained from five or six **(A, B)** experimental repetitions using hPDL-MSCs from a different individual per repetition. For qPCR **(A, B)**, two technical replicates were used, whereas one technical replicate was used per experimental group for PD-L1 and PD-L2 immunostaining **(A, B)**. Each experimental repetition is represented as an individual line **(A, B)**. The Friedman test, followed by the Wilcoxon test, was used for pairwise comparison. * p-value < 0.05 were considered to be significantly increased compared to unstimulated hPDL-MSCs. # p-value < 0.05 were considered to be significantly decreased compared to cytokine-stimulated hPDL-MSCs in the absence of 25(OH)D_3_.

**Figures 4**, **5** show the effects of different 25(OH)D_3_ concentrations on the gene and protein expression of soluble (**Figures 4A–C**) and membrane-bound (**Figures 5A, B**) immunomediators in hPDL-MSCs. We have observed that 25(OH)D_3_ inhibits the cytokine-induced expression of IDO-1 (**Figure 4A**), TSG-6 (**Figure 4B**), PTGS-2/PGE_2_ (**Figure 4C**), PD-L1 (**Figure 5A**), and PD-L2 (**Figure 5B**) in a concentration-dependent manner. This effect was independent of the cytokine type. In the presence of TNF-α a significant reduction started at 1nM 25(OH)D_3_ for IDO-1 (**Figure 4A**) and TSG-6 (**Figure 4B**), at 10nM 25(OH)D_3_ for PTGS-2 (**Figure 4C**) and PD-L2 (**Figure 5B**) and at 100nM for PD-L1 (**Figure 5A**). A significant decline in IL-1β-induced gene expression of TSG-6 (**Figure 4B**) started at 1nM 25(OH)D_3_ and of IDO-1 (**Figure 4A**), PTGS-2 (**Figure 4C**) and PD-L1 (**Figure 5A**) at 100nM 25(OH)D_3_.

A significant reduction of TNF-α-induced IDO-1 protein expression (**Figure 4A**) started at 10nM 25(OH)D_3_, whereas TSG-6 (**Figure 4B**), PD-L1 (**Figure 5A**) and PD-L2 (**Figure 5B**) protein expression and the production of PGE_2_ (**Figure 4C**) were significantly reduced at 100nM. IL-1β-triggered IDO-1 protein expression (**Figure 4A**) significantly decreased from 10nM. The effect of 25(OH)D_3_ on IL-1β-stimulated TSG-6 protein expression (**Figure 4B**) and PGE_2_ (**Figure 4C**) caused no significant decline due to high inter-donor variability. A significant reduction was also observed for IL-1β-induced PD-L1 (**Figure 5A**) and PD-L2 (**Figure 5B**) expression at 100nM 25(OH)D_3_.

Additionally, TNF-α and IL-1β induced IDO-1 enzymatic activities (**Figure 4A**) were significantly decreased from 10 to 100nM 25(OH)D_3_, and at 1 and 100nM 25(OH)D_3_, respectively. Together, these data hint at the potential of 25(OH)D_3_ to reduce the immunomodulatory activities of hPDL-MSCs by suppressing the cytokine-boosted production of various immunomediators.

### 3.4 Pharmacological inhibition of IDO-1 influences the hPDL-MSCs mediated effects of 25(OH)D_3_ on CD4^+^ T lymphocytes

**Figure 6:**
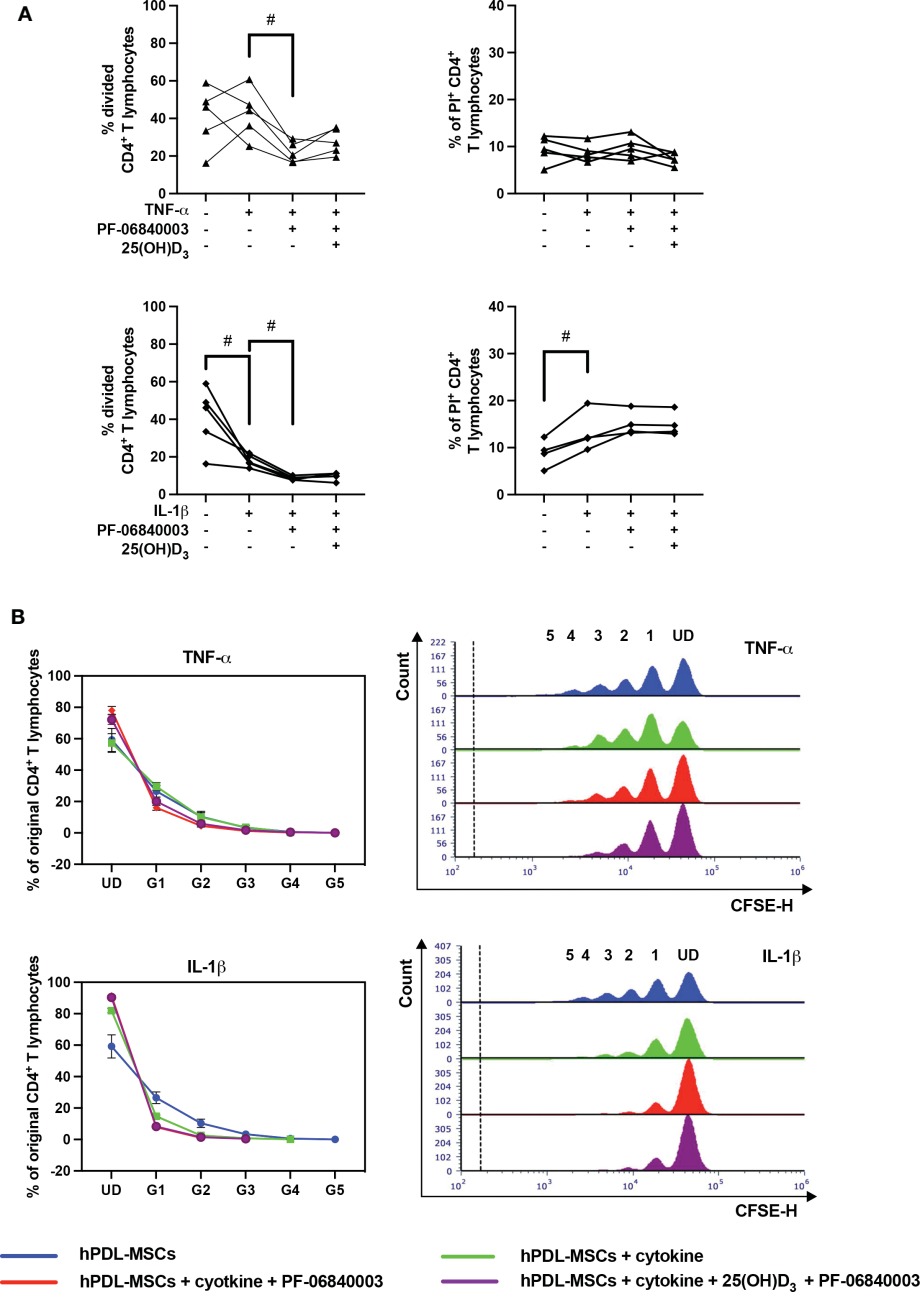
Influence of IDO-1 pharmacological inhibition on the CD4^+^ T lymphocyte proliferation and viability in the presence of hPDL-MSCs under different cytokine conditions. After five days of indirect co-culture with hPDL-MSCs, flow cytometry analysis was used to determine the proliferation and viability of CD4^+^ T lymphocytes by CFSE and PI staining, respectively. **(A)** reveals the percentage of original CD4^+^ T lymphocytes that are divided and the percentage of PI^+^ CD4^+^ T lymphocytes, whereas **(B)** shows the number of original CD4^+^ T lymphocytes as a percent of the total number of original cells for each single cell generation (UD = undivided; G1-G5 = generation 1 – generation 5). Additionally, representative histograms show the number of CD4^+^ T lymphocytes for all generations (UD and 1-5). Data were obtained from five **(A, B)** or four **(A)** experimental repetitions using hPDL-MSCs from a different individual per repetition. One technical replicate was used per experimental group. In **(A)**, each experimental repetition is represented as an individual line. The percentage of original CD4^+^ T lymphocytes in **(B)** are presented as mean ± S.E.M. The Friedman test, followed by the Wilcoxon test, was used for pairwise comparison **(A)**. Viability data of CD4^+^ T lymphocytes, which were co-cultured with IL-1β-treated hPDL-MSCs, were analyzed using one-way ANOVA and Tukey’s multiple comparison test. # p-value < 0.05 were considered to be statistically significant between groups as indicated.

**Figure 6** shows the effects of IDO-1 inhibition on the proliferation and viability of CD4^+^ T lymphocytes in the indirect co-culture model. IDO-1 inhibition significantly counteracted the effect of TNF-α-treated hPDL-MSCs on the % of divided CD4^+^ T lymphocytes (**Figure 6A**), also showing a decreased and increased % of original CD4^+^ T lymphocytes in formed and undivided generations, respectively (**Figure 6B**). On the contrary, IDO-1 inhibition significantly augmented the inhibitory effect of IL-1β-treated hPDL-MSCs, with a reduced % of divided (**Figure 6A**) and original CD4^+^ T lymphocytes in the decreased number of formed generations (**Figure 6B**). If IDO-1 was pharmacologically inhibited, 25(OH)D_3_ caused an increase in CD4^+^ T lymphocyte proliferation independently from the cytokine type but without statistical significance (**Figure 6A**). IDO-1 inhibition caused no changes in the effect of 25(OH)D_3_ on the percentage of non-viable CD4^+^ T lymphocytes independently of the present cytokine type (**Figure 6A**).

**Figure 7:**
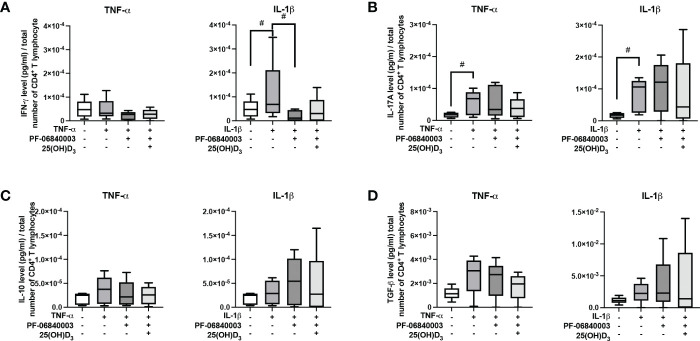
Influence of IDO-1 pharmacological inhibition on the production of IFN-γ, IL-17A, IL-10, and TGF-β in CD4^+^ T lymphocytes in the presence of hPDL-MSCs under different cytokine conditions. After five days of indirect co-culture with hPDL-MSCs, the concentrations of IFN-γ **(A)**, IL-17A **(B)**, IL-10 **(C)**, and TGF-β **(D)** were measured in conditioned media using appropriate ELISAs and the total CD4^+^ T lymphocyte numbers per sample were determined using a cell counting chamber. Measured cytokine levels (pg/ml) were normalized to the total CD4^+^ T lymphocyte number. Data were obtained from five **(A-D)** experimental repetitions using hPDL-MSCs from a different individual per repetition. One technical replicate was used per experimental group. The normalized cytokine levels were presented as a box-whisker plot showing the minimum and maximum values **(A-D)**. The Friedman test, followed by the Wilcoxon test, was used for pairwise comparison. # p-value < 0.05 were considered to be statistically significant between groups as indicated.

**Figure 7** shows the effect of IDO-1 inhibition on the production of pro- and anti-inflammatory cytokines in the indirect co-culture model. In the presence of an IDO-1 inhibitor, the increasing effect of IL-1β-triggered hPDL-MSCs on IFN-γ was significantly counteracted (**Figure 7A**). No significant effects of 25(OH)D_3_ were detected during IDO-1 inhibition (**Figure 7**).

All these data suggest that IDO-1 seems to be partly involved in the hPDL-MSCs-based effects of 25(OH)D_3_ on CD4^+^ T lymphocytes because many of the hPDL-MSCs-mediated effects of 25(OH)D_3_ on cytokine production by CD4^+^ T lymphocytes were not detected when IDO-1 was inhibited.

### 3.5 Pharmacological inhibition of PTGS-2 influences the hPDL-MSCs mediated effects of 25(OH)D_3_ on CD4^+^ T lymphocytes

**Figure 8:**
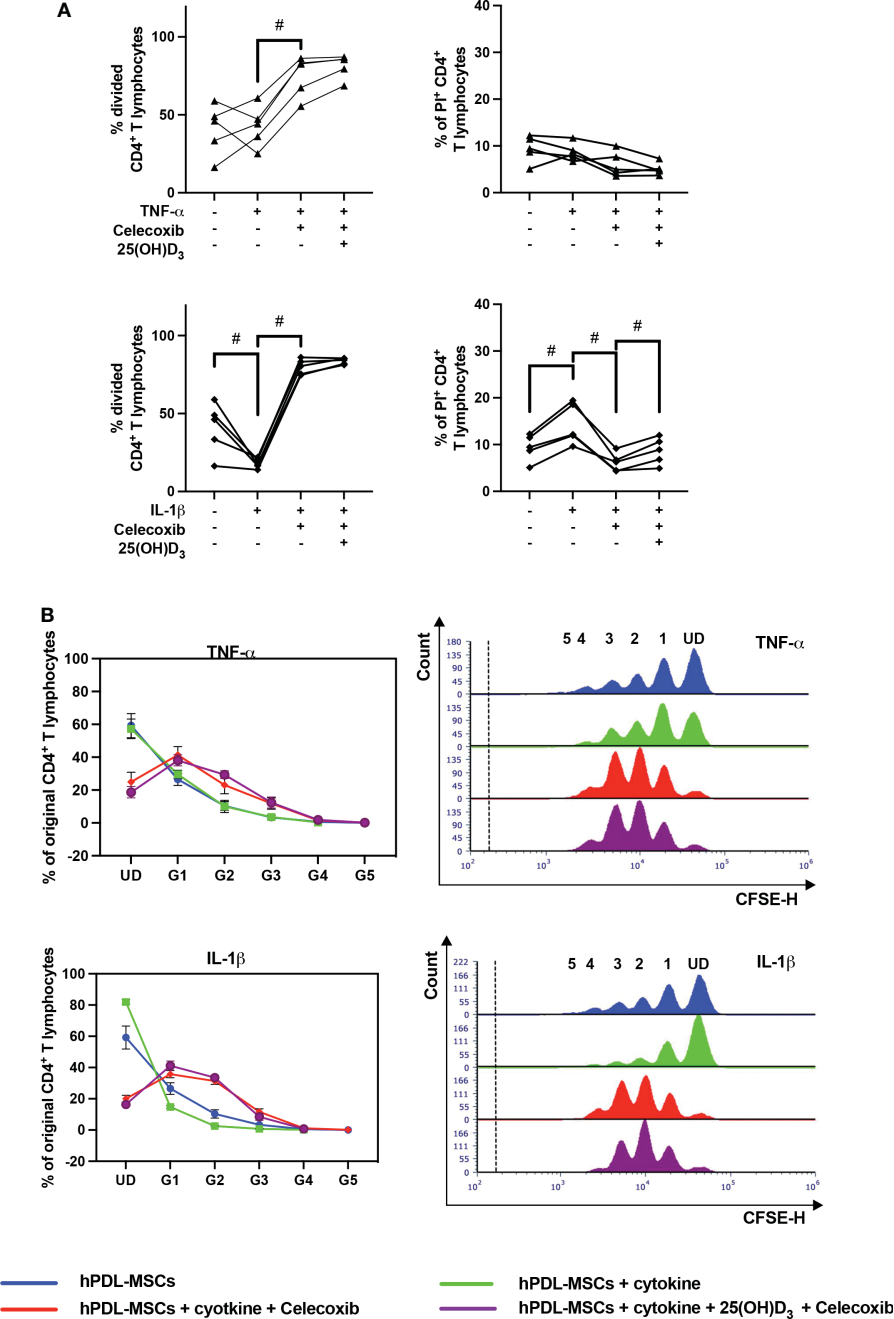
Influence of PTGS-2 pharmacological inhibition on the CD4^+^ T lymphocyte proliferation and viability in the presence of hPDL-MSCs under different cytokine conditions. After five days of indirect co-culture with hPDL-MSCs, flow cytometry analysis was used to determine the proliferation and viability of CD4^+^ T lymphocytes by CFSE and PI staining, respectively. **(A)** reveals the percentage of original CD4^+^ T lymphocytes that have divided and the percentage of PI^+^ CD4^+^ T Lymphocytes, whereas **(B)** shows the number of original CD4^+^ T lymphocytes as a percent of the total number of original cells for each single cell generation (UD = undivided; G1-G5 = generation 1 – generation 5). Additionally, representative histograms show the number of CD4^+^ T lymphocytes for all generations (UD and 1-5). Data were obtained from five **(A, B)** experimental repetitions using hPDL-MSCs from a different individual per repetition. One technical replicate was used per experimental group. In **(A)**, each experimental repetition is represented as an individual line. The percentage of original CD4^+^ T lymphocytes in **(B)** are presented as mean ± S.E.M. The Friedman test, followed by the Wilcoxon test, was used for pairwise comparison **(A)**. # p-value < 0.05 were considered to be statistically significant between groups as indicated.

**Figure 8** shows the effects of PTGS-2 inhibition on the proliferation and viability of CD4^+^ T lymphocytes in the indirect co-culture model. PTGS-2 inhibition significantly increased the percentage of divided CD4^+^ T lymphocytes in the presence of TNF-α-treated hPDL-MSCs (**Figure 8A**). The effect of IL-1β-treated hPDL-MSCs was significantly counteracted by PTGS-2 inhibition. Additionally, these conditions caused a decrease and an increase in the % of original CD4^+^ T lymphocytes of the undivided and the formed generations, respectively (**Figure 8B**). PTGS-2 inhibition significantly counteracted the effect of IL-1β-treated hPDL-MSCs on the percentage of non-viable CD4^+^ T lymphocytes (**Figure 8A**). No significant effect of 25(OH)D_3_ on CD4^+^ T lymphocyte proliferation during PTGS-2 inhibition was observed (**Figures 8A, B**). However, 25(OH)D_3_ significantly increased the percentage of non-viable CD4^+^ T lymphocytes in the presence of IL-1β and PTGS-2 inhibition (**Figure 8A**).

**Figure 9:**
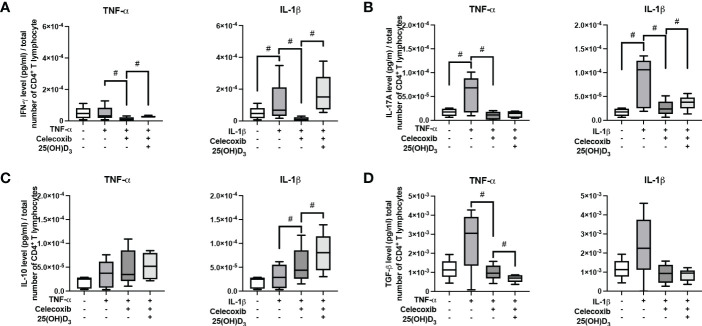
Influence of PTGS-2 pharmacological inhibition on the production of IFN-γ, IL-17A, IL-10, and TGF-β in CD4^+^ T lymphocytes in the presence of hPDL-MSCs under different cytokine conditions. After five days of indirect co-culture with hPDL-MSCs, the concentrations of IFN-γ **(A)**, IL-17A **(B)**, IL-10 **(C)**, and TGF-β **(D)** were measured in conditioned media using appropriate ELISA and the total CD4^+^ T lymphocyte numbers per sample were determined using a cell counting chamber. Measured cytokine levels (pg/ml) were normalized to the total CD4^+^ T lymphocyte number per group. Data were obtained from five **(A–D)** experimental repetitions using hPDL-MSCs from a different individual per repetition. One technical replicate was used per experimental group. The normalized cytokine levels were presented as a box-whisker plot showing the minimum and maximum values **(A–D)**. The Friedman test, followed by the Wilcoxon test, was used for pairwise comparison. # p-value < 0.05 were considered to be statistically significant between groups as indicated.

**Figure 9** shows the effects of PTGS-2 inhibition on the production of pro- and anti-inflammatory cytokines in the indirect co-culture model. In the presence of PTGS-2 inhibitor, the increasing effects of cytokine-treated hPDL-MSCs on IFN-γ (**Figure 9A**), IL-17A (**Figure 9B**), and TGF-β (**Figure 9D**) were significantly counteracted. This was observed under different stimulation conditions. Inhibition of IL-1β-induced PTGS-2 significantly strengthened IL-10 production (**Figure 9C**). In the presence of PTGS-2 inhibitor and IL-1β, 25(OH)D_3_ significantly increased the IFN-γ (**Figure 9A**), IL-17A (**Figure 9B**), and IL-10 levels (**Figure 9C**). Additionally, PTGS-2 inhibition caused an increase in IFN-γ concentration when adding TNF-α and 25(OH)D_3_ (**Figure 9A**). In contrast, the TGF-β concentration was significantly reduced by 25(OH)D_3_ when inhibiting TNF-α-induced PTGS-2 (**Figure 9D**). Together, these results indicate that PTGS-2 is partly involved in the hPDL-MSCs-based effects of 25(OH)D_3_ on CD4^+^ T lymphocytes proliferation and cytokine production.

## 4 Discussion

In an indirect *in vitro* co-culture model, we analyzed the effects of 25(OH)D_3_ on the immunomodulatory activities of hPDL-MSCs towards CD4^+^ T lymphocytes. This model showed that 25(OH)D_3_ significantly counteracted the suppressive effects of IL-1β-treated hPDL-MSCs on CD4^+^ T lymphocyte proliferation, whereas no effects were observed in the presence of TNF-α. Additionally, 25(OH)D_3_ significantly increased the percentage of viable CD4^+^ T lymphocytes *via* TNF-α- or IL-1β-treated hPDL-MSCs. 25(OH)D_3_ also caused a significant decrease in IFN-γ, IL-17A and TGF-β productions, which were triggered by TNF-α-treated hPDL-MSCs. Investigating the impact of 25(OH)D_3_ on the cytokine-induced expression of various immunomediators in hPDL-MSCs, demonstrated a significant decrease in the expression of IDO-1, TSG-6, PTGS-2/PGE_2_, PD-L1, and PD-L2. Inhibiting IDO-1 and PGE_2_ qualitatively changed many of the hPDL-MSCs-mediated effects of 25(OH)D_3_ on CD4^+^ T lymphocyte proliferation, viability, and cytokine production. Together these data indicate that 25(OH)D_3_ affects the immunomodulatory ability of hPDL-MSCs and that this effect and its mechanisms are versatile and depend on the local cytokine environment.

In the absence of any cytokines, 25(OH)D_3_ did not influence the hPDL-MSCs-mediated modulation of the proliferation and the percentage of non-viable CD4^+^ T lymphocytes. However, various effects of 25(OH)D_3_ were observed in the presence of TNF-α- or IL-1β-treated hPDL-MSCs, which are mainly secreted by macrophages and polymorphonuclear leukocytes during the initial phase of periodontal inflammation. This indicates that the immunomodulatory effects of 25(OH)D_3_ on CD4^+^ T lymphocytes are mediated through the cytokine-induced immunomodulatory mechanisms of hPDL-MSCs. Adding TNF-α-stimulated hPDL-MSCs to the co-culture model reduced the percentage of non-viable CD4^+^ T lymphocytes by 25(OH)D_3_, but no significant effect of 25(OH)D_3_ on CD4^+^ T lymphocyte proliferation was observed. In contrast, 25(OH)D_3_ significantly counteracted the immunosuppressive effect of IL-1β-treated hPDL-MSCs on CD4^+^ T lymphocytes, thus facilitating a more pro-inflammatory status. Due to the presence of cytokines in the co-culture setup, the observed effects of cytokine-treated hPDL-MSCs on CD4^+^ T lymphocyte proliferation may also arise from a direct influence of cytokine on CD4^+^ T lymphocytes. In our previous study, we observed no direct effects of IL-1β on CD4^+^ T lymphocyte proliferation (14). Hence, the observed inhibitory effect of IL-1β is exclusively regulated by hPDL-MSCs. In contrast, TNF-α directly inhibited CD4^+^ T lymphocyte proliferation, whereas in the presence of hPDL-MSCs no changes were observed. Together this indicates that the effects of cytokines on CD4^+^ T lymphocytes are modified mainly by hPDL-MSCs (14).

Furthermore, we investigated the secretion of functional cytokines (IFN-γ, IL-17A, IL-4, IL-10 and TGF-β) by CD4^+^ T lymphocytes. IFN-γ is mainly produced by Th1 lymphocytes (46), IL-17A is characteristically secreted by Th17 lymphocytes (47), and IL-10 and TGF-β are expressed by Tregs (48). All three CD4^+^ T lymphocyte subsets play essential roles during periodontitis pathogenesis (46). IL-4, a distinctive cytokine for Th2 lymphocytes, was not detectable. These data also showed the dependency of the 25(OH)D_3_ effects on the cytokine-induced immunomodulatory mechanisms of hPDL-MSCs. Additionally, these data indicate the importance of the present cytokine type. Mainly, TNF-α-treated hPDL-MSCs caused inhibitory effects of 25(OH)D_3_ on the secretion of all cytokines, whereas in the presence of IL-1β-treated hPDL-MSCs, 25(OH)D_3_ caused no significant changes in the secretion of pro- and anti-inflammatory cytokines. Thus, the data on cytokine production, like the proliferation and the percentage of non-viable CD4^+^ T lymphocytes, suggests highly versatile effects of 25(OH)D_3_ on the interaction between hPDL-MSCs and CD4^+^ T lymphocytes.

Next, we aimed to clarify the mechanisms underlying all these 25(OH)D_3_-based changes in the immunomodulatory activities of hPDL-MSCs toward CD4^+^ T lymphocytes. Particularly, we investigated the production of IDO-1, TSG-6, PGE_2_, PD-L1 and PD-L2 (5), since the expression of these immunomediators is known to be induced by TNF-α and IL-1β (12, 43, 49–51). IDO-1 is the rate-limiting enzyme of the tryptophan metabolism, oxidating L-tryptophan into N-formyl-kynurenine, which is further metabolized to L-kynurenine (49). The depletion of L-tryptophan and L-kynurenine itself has a negative influence on various immune cells, such as the proliferation, survival, and differentiation of T lymphocytes (52–55). Although the operating mode of TSG-6 is not fully elucidated, some evidence shows a potential interaction with chemokines, causing a reduction in chemokine availability and changes in the chemokine-chemokine receptor interaction (56, 57). hPDL-MSCs-secreted TSG-6 influences various immune cells, such as adjusting the maturation and cytokine production of macrophages (58–60). PGE_2_ is one of the end-products of arachidonic metabolism and is produced by prostaglandin-endoperoxide synthase (PTGS)-2 (61). *Via* binding to E-type prostanoid receptors, PGE_2_ regulates the functions of various immune cells (62)such as the proliferation, differentiation, and survival of T lymphocytes (52, 53). The transmembrane proteins PD-L1 and PD-L2 interact with their programmed cell death 1/2 (PD-1/2) receptors on the cell membrane of various immune cells. These interactions induce immune cell apoptosis and suppress the proliferation, activation, and cytokine production of various immune cells, such as T lymphocytes (12, 43, 63). Our results mainly showed a decrease in the production of these mediators by 25(OH)D_3_. This suggests a decline in the immunomodulatory activities of hPDL-MSCs and therefore a pro-inflammatory effect of 25(OH)D_3_.

Since in our used indirect co-culture model the soluble immunomediators are of most relevance and IDO-1 and PGE_2_ are mainly involved in the interaction between hPDL-MSCs and CD4^+^ T lymphocytes (4, 63, 64), we investigated the contribution of these two immunomediators to the hPDL-MSCs-mediated effects of 25(OH)D_3_ on CD4^+^ T lymphocytes by pharmacologically inhibiting IDO-1 and PTGS-2 during the co-culture experiments. The effects of 25(OH)D_3_ on CD4^+^ T lymphocyte proliferation and viability were not affected by IDO-1 or PTGS-2 inhibitors in the presence of TNF-α-treated hPDL-MSCs. However, PTGS-2 inhibition abolished the stimulatory effect of 25(OH)D_3_ on CD4^+^ T lymphocyte proliferation and its anti-cell-death effect *via* IL-1β-stimulated hPDL-MSCs. This confirms that 25(OH)D_3_ executes at least its anti-cell-death effect on CD4^+^ T lymphocytes by influencing the IL-1β-induced expression of PTGS-2 in hPDL-MSCs. Decreasing the IL-1β-induced expression of PGE_2_ in hPDL-MSCs by 25(OH)D_3_ seems to reduce the observed number of non-viable CD4^+^ T lymphocytes and hence the cell death-inducing potential of IL-1β-treated hPDL-MSCs.

The inhibition of IDO-1 abolished the inhibitory effect of 25(OH)D_3_ on cytokine production in the co-culture setting in the presence of TNF-α-triggered hPDL-MSCs. PTGS-2 inhibition only partially reversed the repressing effect of 25(OH)D_3_ on the cytokine production in the presence of TNF-α or IL-1β-triggered hPDL-MSCs. Taking this together, the mechanisms of 25(OH)D_3_ effects mediated by hPDL-MSCs depend on the inflammatory environment. Moreover, since not all detected effects of 25(OH)D_3_ were affected by IDO-1 and PTGS-2 inhibition, it is likely that also other immunomediators or mechanisms are involved and should be clarified by further studies.

The data of the present study are further evidence of the versatile immunomodulatory effects of vitamin D_3_ metabolites and their high plasticity depending on hPDL-MSCs and the local cytokine environment. 25(OH)D_3_ shows pro- or anti-inflammatory immunomodulatory mechanisms depending on the present cytokine type. Additionally, 25(OH)D_3_ promotes an anti-inflammatory status by suppressing the production of pro-inflammatory cytokines in hPDL-MSCs under pro-inflammatory conditions (31). At first glance, this plasticity was also observed for 1,25(OH)_2_D_3_. Similar to 25(OH)D_3_, the effects of 1,25(OH)_2_D_3_ on CD4^+^ T lymphocytes depend on the present cytokine type but also on the presence of hPDL-MSCs. In our previous studies, we demonstrated distinct effects of 1,25(OH)_2_D_3_ on CD4^+^ T lymphocyte proliferation in the presence and absence of indirectly co-cultured and cytokine-stimulated hPDL-MSCs. Furthermore, our early study showed a pro-inflammatory effect of 1,25(OH)_2_D_3_ on CD4^+^ T lymphocyte proliferation *via* IFN-γ-treated hPDL-MSCS and its anti-inflammatory effect *via* IL-1β-stimulated hPDL-MSCs (42, 65). In contrast, 25(OH)D_3_ caused an increase in CD4^+^ T lymphocyte proliferation in co-culture with IL-1β-treated hPDL-MSCs. This indicates that hPDL-MSCs-mediated effects of 25(OH)D_3_ and 1,25(OH)_2_D_3_ are at least partially different. We can hypothesize that the observed effects of 25(OH)D_3_ are not exclusively due to the conversion of 25(OH)D_3_ to 1,25(OH)_2_D_3_ by the local vitamin D_3_ metabolism (66). Further, it seems that 25(OH)D_3_ and 1,25(OH)_2_D_3_ execute their indirect immunomodulatory effects *via* different mechanisms in hPDL-MSCs. Although both vitamin D_3_ metabolites decreased the production of investigated immunomediators in hPDL-MSCs, inhibitory experiments revealed an IDO-1 dependency of 1,25(OH)_2_D_3_, in regard to CD4^+^ T lymphocyte proliferation (65), whereas the indirect 25(OH)D_3_ effects on the CD4^+^ T lymphocyte seem to be IDO-1 independent. A potential mechanism could be related to the conversion of 25(OH)D_3_ to 24R,25(OH)_2_D_3_ by CYP24A1 (67). 24R,25(OH)_2_D_3_ possesses biological activity in MSCs (68) and the expression of CYP24A1 could be induced through the activation of vitamin D receptor by 1,25(OH)_2_D_3_ (69). However, this assumption should be investigated by further studies.

By extrapolating our results into a clinical context, we can conclude that both vitamin D_3_ metabolites – 1,25(OH)_2_D_3_ (42, 65) and 25(OH)D_3_ – exhibit a high grade on immunomodulatory plasticity with which they fine-tune the periodontal tissue homeostasis. It seems that not only 1,25(OH)_2_D_3_ (42, 65), but also 25(OH)D_3_ is involved in regulating the balance between activating and resolving periodontal tissue inflammation during periodontitis, which is orchestrated by various tissue-resident cells, such as immune cells and hPDL-MSCs (70). Similar to 1,25(OH)_2_D_3_ (42, 65), the influence of 25(OH)D_3_ on this balancing act seems to be highly dependent on the local microenvironment. Although several animal studies demonstrated anti-inflammatory effects of 25(OH)D_3_ by diminishing periodontal inflammation (36–38), the results of our studies (42, 65) showed that the immunomodulatory effects of the two vitamin D_3_ metabolites have a higher complexity on the cellular level. Hence, this complexity should be considered when using vitamin D_3_ supplementation for inflammatory diseases, such as periodontitis. Based on this study, giving periodontologists exact advice in vitamin D_3_ supplementation is not possible, due to the study’s *in vitro* character. Our data imply that the generally assumed anti-inflammatory ability of vitamin D_3_ (16, 17) might be diminished or even abolished in the inflammatory environment. Therefore, its clinical effectiveness might depend on the presence of inflammation, and it should be prescribed at a certain time point of the therapy, but animal and clinical studies should prove this assumption.

However, translating these data into a clinical setting has several limitations. Although the complex indirect *in vitro* co-culture model considers the reciprocal interaction between CD4^+^ T lymphocytes and hPDL-MSCs, the model only allows paracrine interactions, excluding direct cell-to-cell contact mechanisms, which also execute essential immunomodulatory functions *in vivo* (4, 12). Additionally, the purified immune cell populations show 93% CD4^+^ T lymphocytes. The contamination with other PBMCs may cause confounder-related effects. Further, the contribution of hPDL-MSCs to the inflammatory status of the periodontal tissue depends on regulating cells of both the innate and adaptive immune systems rather than on a single immune cell subset (5). Also, culturing hPDL-MSCs *in vitro* results in changes in their phenotype as well as functional capabilities. To mimic the *in vivo* situation more accurately, 3D PDL-cementum-like approaches (71) could be used. Furthermore, the exclusive use of 25(OH)D_3_ does not reflect the complex situation *in vivo* with the presence of 1,25(OH)_2_D_3_ and its immunoregulatory capability (35). Additionally, our *in vitro* model does not consider the capacity of hPDL-MSCs to metabolize 25(OH)D_3_ to 1,25(OH)_2_D_3_ under basal and pro-inflammatory conditions (72–75). This can be overcome by investigating the local vitamin D_3_ metabolism in the PDL under different inflammatory conditions and by using hPDL-MSCs in our *in vitro* co-culture model with a depletion of the enzyme which is involved in converting 25(OH)D_3_ to 1,25(OH)_2_D_3_.

## 5 Conclusion

In conclusion, this *in vitro* study demonstrated the modulating potential of the vitamin D_3_ precursor, 25(OH)D_3_, on the cytokine-dependent immunomodulatory activities of hPDL-MSCs toward CD4^+^ T lymphocytes, for the first time. Although *in vivo* studies showed anti-inflammatory properties (36–38), our study exhibited high plasticity of these indirect immunomodulatory effects of 25(OH)D_3_. Like 1,25(OH)_2_D_3_ (42, 65), these activities highly depend on the presence of inflammatory cytokines and the cytokine type. Together with the already known high plasticity of the immunomodulatory potential of 1,25(OH)_2_D_3_ (31, 42, 65), these two vitamin D_3_ metabolites seem to be highly potent regulators of periodontal tissue homeostasis. In further studies, it is necessary to elucidate the exact cellular functions of 25(OH)D_3_ during periodontal tissue inflammation, such as during periodontitis.

## Data availability statement

The raw data supporting the conclusions of this article will be made available by the authors, without undue reservation.

## Ethics statement

The studies involving human participants were reviewed and approved by Ethics Committee of the Medical University of Vienna. The patients/participants provided their written informed consent to participate in this study.

## Author contributions

Conceptualization, CB, OA, and AB. Methodology, CB and JG. Validation, CB and OA. Formal analysis, CB, OA, AB, and JG. Resources, OA, XR-F, and AM. Writing-original draft preparation, CB. Writing-review and editing, AB, XR-F, AM, and OA. Supervision, OA, XR-F, and AM. Project administration, OA. All authors contributed to the article and approved the submitted version.
