# Supplementary material for: 25-hydroxyvitamin D3 generates immunomodulatory plasticity in human periodontal ligament-derived mesenchymal stromal cells that is inflammatory context-dependent
Source: Front Immunol. 2023 Jan 24;14:1100041. doi: 10.3389/fimmu.2023.1100041 (PMC9902380; doi:10.3389/fimmu.2023.1100041)
Supplement: Supplementary file 1 [file DataSheet_1.docx]

Supplementary Material

# Supplementary Figures

**Supplementary Figure 1. Representative dot plots and histograms show the flow cytometry gating strategy for CD4^+^ T lymphocyte proliferation and viability analysis.** Proliferation and the percentage of non-viable CD4^+^ T lymphocytes were analyzed after co-culture with hPDL-MSCs. The histogram's green area and the dotted line represent the distinct CD4^+^ T lymphocyte generation and the staining background, respectively.
